# Photodynamic Efficiency: From Molecular Photochemistry to Cell Death

**DOI:** 10.3390/ijms160920523

**Published:** 2015-08-31

**Authors:** Isabel O. L. Bacellar, Tayana M. Tsubone, Christiane Pavani, Mauricio S. Baptista

**Affiliations:** 1 Instituto de Química, Universidade de São Paulo, São Paulo 05508-900, Brazil; E-Mails: isabel@iq.usp.br (I.O.L.B.); tayana_tmt@hotmail.com (T.M.T.); 2 Programa de Pós Graduação em Biofotônica Aplicada às Ciências da Saúde, Universidade Nove de Julho, São Paulo 01504-001, Brazil; E-Mail: chrispavani@uninove.br

**Keywords:** photodynamic therapy, photosensitization, photooxidation, cell death, subcellular localization

## Abstract

Photodynamic therapy (PDT) is a clinical modality used to treat cancer and infectious diseases. The main agent is the photosensitizer (PS), which is excited by light and converted to a triplet excited state. This latter species leads to the formation of singlet oxygen and radicals that oxidize biomolecules. The main motivation for this review is to suggest alternatives for achieving high-efficiency PDT protocols, by taking advantage of knowledge on the chemical and biological processes taking place during and after photosensitization. We defend that in order to obtain specific mechanisms of cell death and maximize PDT efficiency, PSes should oxidize specific molecular targets. We consider the role of subcellular localization, how PS photochemistry and photophysics can change according to its nanoenvironment, and how can all these trigger specific cell death mechanisms. We propose that in order to develop PSes that will cause a breakthrough enhancement in the efficiency of PDT, researchers should first consider tissue and intracellular localization, instead of trying to maximize singlet oxygen quantum yields in *in vitro* tests. In addition to this, we also indicate many open questions and challenges remaining in this field, hoping to encourage future research.

## 1. Introduction

Photodynamic therapy (PDT) relies on the combination of a photosensitizer (PS), light, and oxygen [O_2_(^3^Σ_g_^−^)] to eliminate unwanted cells. These cells can be tumor cells or microorganisms, such as fungi and bacteria. The main agent in PDT is the triplet excited state of the PS, which can sensitize the formation of singlet oxygen [O_2_(^1^Δ_g_)] or radicals. These reactive species are responsible for damaging biomolecules and thus promoting cell death, which is the desired outcome of PDT [1,2,3,4,5]. If compared to other treatments, PDT has several advantages. The photo-activation allows for localized action, reducing the side effects of PDT, which can also be applied in conjunction with other clinical modalities (e.g., post-surgery). In addition, PDT has multiple cellular targets and, therefore, is not believed to lead to drug resistance. PDT is also potentially suitable to public health systems, since combination of low cost PSes and light sources turns it into an affordable treatment [4,6,7,8]. However, in spite of these advantages and the growing knowledge on the efficiency of PDT, it is evident that this clinical modality is still not widespread. This can be attributed in part to a lack of knowledge on some of the molecular mechanisms taking place on PDT. Notably, it is still unclear which are the most important biological targets of photooxidation reactions and, hence, which are the most effective strategies to achieve cell death. Starting at the molecular level and progressing to the biological outcomes, we discuss herein some factors that should be considered when aiming to a successful PDT effect, taking into account that PDT mechanisms depend both on the PS and also on the interaction of the PS with the nanoenvironment where it is. Next, we present some aspects that are still missing to create a clear mechanistic view of PDT and pinpoint possible approaches to overcome the challenges remaining on this field. We believe that this analysis may help to understand which factors should be considered when developing an improved PDT protocol and also reinforces the need of tailor-made PDT approaches.

## 2. Biological Targets of Photooxidations

### 2.1. Photooxidation of Biomolecules

**Figure 1:**
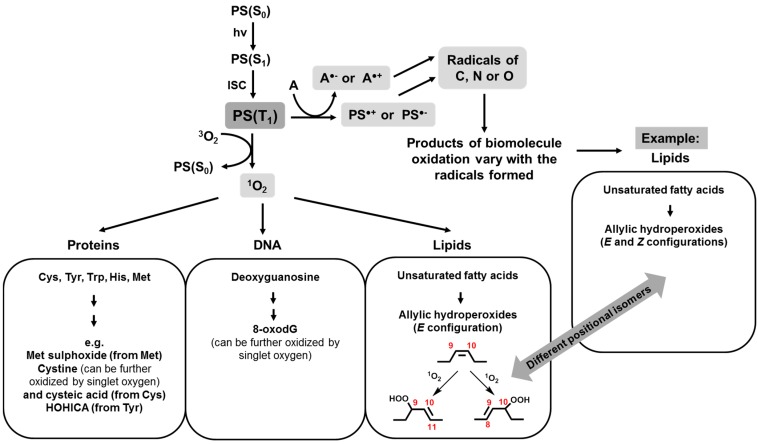
Main routes and initial products of singlet oxygen and radical mediated photooxidations (for details on the products formed, refer to citations in the main text). Abbreviations: PS: photosensitizer; ISC: intersystem crossing; A: substrate for electron transfer reactions.

The first step in the photoinduced process is light absorption. Once in a singlet excited state, PS molecules can be converted to a triplet excited state by intersystem crossing (ISC). Any structural features in a molecule favoring spin-orbit coupling (such as heavy atoms) favor this process. ISC is a fast (sub-picosecond), adiabatic, and, hence, non-radiative transition, between states with different multiplicities. The crossing between potential energy surfaces of the singlet and triplet excited states allows this otherwise spin-forbidden process to occur. However, this same process is much less probable for the decay of the triplet excited state to the ground state (singlet), accounting for the longer lifetime of triplet excited states [9]. This longer lifetime increases the probability that it interacts with molecules nearby, leading to energy or electron transfer (Figure 1). It is important to remark that in many cases these interactions will simply result in deactivation of the triplet excited state without inducing further chemical reactions. An example is quenching by an initial electron transfer reaction, followed by back-electron transfer, thus recovering the ground state PS. From a biological point of view, these processes can be regarded as dead ends, since no chemical changes arise. Of course, light is converted into heat and there are some therapies aiming for killing cells with the excess of local heat [10,11,12]. The important step in the induction of tissue damage occurs when the triplet excited state sensitizes the formation of reactive species, which can lead to biomolecule photooxidation. It is possible to separate two main pathways: (i) energy transfer to oxygen forming singlet oxygen, which is an electrophilic agent that can directly add to double bonds; (ii) electron transfer reactions of the PS with biological substrates, forming radicals that can initiate chain reactions. Many kinds of substrates can partake in electron transfer process, leading to formation of different types of radicals (centered both on the PS and on the substrate) [3]. Initially, semi-oxidized or semi-reduced radicals are formed, and these will usually suffer further reactions. The semi-reduced PS radical can react with electron acceptors, such as oxygen (see further discussion below), regenerating the PS. However, radicals can divert the final product of the reaction to a stable species different from the original PS, like a different chromophore or even a species that does not absorb light. Strictly speaking, these reactions would not fit in the definition of photosensitization, which implies the recovery of the PS after it absorbs light and produces a photochemical or photophysical change in a second species [13]. The chemical transformations after the electron transfer deactivation of the triplets will be further discussed below.

Oxidation reactions involving singlet oxygen can have different consequences. Due to its unoccupied π*_2p_ orbital, singlet oxygen is highly reactive towards electron-rich compounds. For unsaturated compounds, different kinds of reactions can occur depending on the substrate, such as Diels-Alder reaction producing endoperoxides from 1,3-dienes, 1,2-cycloaddition, to electron rich alkenes forming dioxetanes, and hydroperoxide formation by ene reaction with alkenes containing allylic hydrogens. This latter reaction is well known for leading to lipid oxidation, yielding lipid hydroperoxides, which are mainly in the *E* (*trans*) configuration [14]. The formation of *trans* double bonds in lipids can have some consequences to the membranes, which usually bare only *cis*-configuration lipids, but this effect has not been considered by our community yet. These hydroperoxides are unstable in the presence of radicals, high temperatures or transition metals, starting a peroxidation chain reaction [3,15,16]. Singlet oxygen can also react with molecules containing nitrogen or sulfur, like some amino-acids and nitrogenous bases. Tryptophan, tyrosine, histidine, methionine, cysteine, and cystine are the amino-acids that are oxidized at significant rates by singlet oxygen at physiological pH, forming mainly hydroperoxides and endoperoxides. It is noteworthy that, in order to suffer oxidation, these residues must be exposed to singlet oxygen. This does not always happen when amino acids are within a polypeptide chain and proteins with tertiary structure [3,15,17,18]. As for DNA, oxidation occurs primarily at deoxyguanosine sites, forming an unstable endoperoxide produced through a Diels-Alder 1,4-cycloaddition of singlet oxygen, that ends up forming 8-oxodG, which can be further oxidized by singlet oxygen [19,20,21]. Carbohydrates are believed to be less reactive towards singlet oxygen, but literature on oxidation of carbohydrates in the sole presence of singlet oxygen is still scarce.

For redox reactions there is a greater diversity of reactive species (many of them being able to interconvert between each other). The reactions involved in radical pathways yield a broader range of products and some of them even cause destruction of the PS. Although both radicals and singlet oxygen can attack PSes, causing its degradation, a usual concern about electron transfer reactions is the possibility of increasing this process, since a second electron transfer reaction must take place to regenerate the ground state PS. In fact, the majority of the PSes in use today aim for singlet oxygen generation and not so much for electron transfer reactions. However, it is clear today that these electron-transfer processes in the presence of biological substrates substantially impact the final biological consequences of PDT [22]. Photoinduced generation of radicals can start either with reduction or oxidation of substrates by the triplet excited state of the PS, or even by singlet excited states in the situation when these species are physically attached to biological targets. One should remember that excited states are usually stronger oxidizing and reducing species than their respective ground states [23]. This initial electron (or hydrogen, H^+^ + *e*^−^) transfer step produces radicals (centered in the PS and in the substrate) that can also attack other molecules and create a radical chain reaction. In both cases (*i.e.*, initial reduction or oxidation of the substrate) reaction with dissolved oxygen in subsequent steps leads to overall biomolecule oxidation. The radical chain reaction usually continues long after the irradiation is interrupted and termination only occurs by depletion of reactants (biomolecules or oxygen) or by action of antioxidants [3,16].

In biological conditions, vitamins and reduced coenzymes can act as initial electron donors to the triplet excited state. Riboflavin triplet excited state, for example, is known to react with folate and pyridoxal phosphate [24,25]. However, their oxidized products can be stable and not always induce a radical chain reaction. Amino-acids and proteins, unsaturated lipids, and nitrogenous bases are other possible substrates of reaction with the triplet excited state of PSes [26,27,28]. Incidentally, electron transfer can also happen between two molecules of PS, which is the case in the dye–dye mechanism of PS aggregates. Although direct electron transfer to oxygen is believed to seldom occur, semi-reduced radicals formed in the initial step can reduce oxygen in one-electron steps. This first yields a superoxide radical, which can be further reduced and protonated, yielding hydrogen peroxide. Superoxide dismutase (SOD) can also catalyze the conversion of superoxide radical to hydrogen peroxide in the biological medium. In the presence of ferrous ions, a Fenton reaction can take place, leading to hydroxyl radical production. This species can oxidize most of the biomolecules, generating more complex radicals (e.g., carbon-centered lipid radicals) [3,16].

The only difference between PDT-triggered radical-mediated photooxidations and other kinds of radical-mediated oxidations normally occurring in cells is the initial burst of radicals that is produced by light absorption in the case of PDT. After this step that forms the primary products, reactions will follow pathways unspecific to PDT, yielding several secondary products. Of course the situation is a bit more complicated because there is continuous pumping of PS to the triplet excited state and, thus, continuous formation of radicals and singlet oxygen. It is fair to say that the initial steps in the photooxidation reactions of PDT are a lot better characterized than the progress reactions. These latter involve such a large range of possibilities that nowadays it is impossible to study them in the real biological scenario. It is important to mention that recently developed “omics” tools may change this scenario in the near future [29,30,31,32].

Most studies of photosensitized radical chain reactions are focused on proteins, lipids, carbohydrates, and DNA, though other biologically-relevant molecules can also be oxidized (including natural antioxidants, such as tocopherol). There are many reviews covering these processes extensively [33]. Take the case of lipids, for example: the radical chain reaction usually starts by abstraction of an allylic hydrogen by a radical, resulting in a carbon-centered radical. The ease of formation of this radical increases for polyunsaturated fatty acids containing bis-allylic hydrogens, and decreases for saturated lipids. Oxygen can react with this radical, generating a peroxyl radical. This can either abstract a hydrogen atom from another lipid, yielding a lipid hydroperoxide and a new lipid radical, or follow other reaction pathways that ultimately lead to the formation of oxidized lipids belonging to different organic functions (such as aldehydes, carboxylic acids, alcohols, ketones, and even more complex functions for lipids bearing higher numbers of double bonds). It is noteworthy that radical oxidation of lipids yield both *E* (*trans*) and *Z* (*cis*) hydroperoxides, as well as being able to generate different positional isomers if compared to singlet oxygen oxidation [14,16]. The important message is that products of oxidation by singlet oxygen or radicals may not be the same.

In fact, the existing differences between the singlet oxygen and radical-generated products can be exploited to identify the reactions pathways taking place. Hydroperoxides generated by the oxidation of cholesterol, for example, can be used as biomarkers, since the reaction with singlet oxygen generates mainly 3β-hydroxy-5α-cholest-6-ene-5-hydroperoxide (and minor quantities of 3β-hydroxycholest-4-ene-6α-hydroperoxide and 3β-hydroxycholest-4-ene-6β-hydroperoxide), whereas 3β-hydroxycholest-5-ene-7α-hydroperoxide and 3β-hydroxycholest-5-ene-7β-hydroperoxide are the main products of radical chemistry [16]. However, many molecules commonly considered as biomarkers can be formed by both pathways (e.g., in the former example, some of the products generated by radicals can arise from rearrangements of products formed by singlet oxygen) or by secondary reactions, posing the need of using other methods in conjunction.

In the context of radical reactions, one electron reduction potentials can be used to infer whether a reaction is thermodynamically favored or not. Indeed, a hydroxyl radical has a very high reduction potential (E_0_′ = +2.3 V), being able to oxidize most substrates. However, this data is rarely available for the conditions found in the intracellular environment and also do not foresee other types of reactions and kinetic effects [34]. The occurrence of reactions will also depend on the substrates that the radical encounters. The diffusion distance of radicals is dependent on their reactivity, since more reactive radicals react earlier and do not diffuse along great distances. This is the case of hydroxyl radical, whose reaction rate is diffusion controlled. On the other hand, superoxide radical is a poorly reactive species, thus travelling greater distances before being consumed. In fact, superoxide radicals are known to act as messengers in cell signaling pathways and rarely are the radicals that will cause the final damage. The main cause of damage due to the overproduction of superoxide radicals is usually the possible formation of hydroxyl radicals [33,35].

Singlet oxygen lifetime inside cells will depend on the nanoenvironment where it is formed. In lipid membranes, for example, due to its physical deactivation pathways singlet oxygen can live about 3–4 times longer than in water. Moreover, the high concentration of molecules inside the cell can favor chemical and physical quenching mechanisms, further lowering its lifetime in some nanoenvironments [36,37,38]. For this reason, singlet oxygen diffusion distance inside cells is certainly no longer than 100 nm. When this maximum diffusion distance is compared with the dimension of mammalian cells (diameters in the order of 10–30 μm) or intracellular organelles (for example, mitochondria are 500 nm wide) it is clear that singlet oxygen do not diffuse long enough to act in sites other than its site of generation [39].

Singlet oxygen can be detected by its phosphorescence at 1270 nm. There is no other biologically relevant species known that emits in this wavelength region, allowing spectrally-resolved luminescence measurements to be used as a signature [38,40]. The identity of singlet oxygen is usually further confirmed by solvent effects in isotropic media (especially the lifetime enhancement in deuterated solvents). In isotropic solution, exchanging water to deuterium oxide will certainly increase singlet oxygen lifetime (from 3–4 to 50 μs). However, in most biological scenarios the increase is not as much as expected and the effect is less clear [41,42]. Singlet oxygen lifetime can also be used to infer the environment where it is deactivated. For example, singlet oxygen lifetime was shown to vary in human hair, with a higher lifetime for blond (3.7 μs) than for black hair (0.8 μs). These results were correlated to the amount of melanin in the hair, which is a known quencher of singlet oxygen. Additionally, when samples were measured in different solvents, lifetimes were shorter than expected, showing that singlet oxygen is generated and deactivated within the hair structure [41].

Quenching of singlet oxygen and radicals by specific molecules can also be used to infer which of these species are prevailing in a specific PDT scenario. Sodium azide and carotenoids are known to quench singlet oxygen, while mannitol and BHT suppress radicals [15,16,38]. However, quenching will be affected by concentrations (species and quencher) and intracellular location, and can also be unspecific. For example, the effects of partition also play an important role here, for a hydrophilic quencher may not efficiently suppress reactions taking place on membranes [43]. Another possibility to achieve this is the use of probes that exhibit absorption or emission spectral changes upon reacting with radicals or singlet oxygen. However, these probes usually face the same problem as quenchers, often being unspecific (even among different radicals) and leading to erroneous conclusions [44,45]. Consequently, the prevailing recommendation is to combine as much methods as possible to identify the chemical pathways taking place after light absorption by the PS.

### 2.2. Consequences of Biomolecule Oxidation

Defining strategies to maximize the outcome of the photooxidation reactions is a key point towards the improvement of PDT protocols. The analysis considering the rate constant of reactions between singlet oxygen and biological substrates and their respective intracellular concentrations can result in the very simplistic conclusion that proteins, which are the main components of cells by weight (water excluded), are the most important targets of PDT in the intracellular environment [17,46]. However, this assumption ignores that cells are heterogeneous and that singlet oxygen may be generated closer to other kinds of substrates, which is determined by PS affinity to biomolecules as well as other factors controlling PS subcellular localization. Certainly, many proteins have affinity for PSes, but most PSes bind to membranes and, in agreement, higher lipophilicity is often correlated with increased photodynamic efficiency [47,48,49]. Moreover, lipid membranes have higher concentration of oxygen than the surrounding solution, also favoring quenching of the triplet excited state by oxygen [50,51,52,53]. Actually, different kinds of proteins probably are exposed to different amounts of singlet oxygen (e.g., membrane proteins *vs.* proteins present in the cytosol). Hence, the prediction of the major reactants is not straightforward and cannot be generalized to whole classes of biomolecules, being important to study particular targets in detail. These same considerations should also be valid for radicals, with the additional complication of the larger variety of reactive species.

Since we are still missing analytical tools that allow detailed characterization of biomolecule oxidation products of PDT in biological environments, most studies focus on products that are characterized in *in vitro* experimental systems, based mainly on the chemical reactivity of singlet oxygen. In some cases, however, the consequences of the oxidation of specific kinds of biomolecules can be predicted based on structural and physicochemical considerations and be later validated on biological systems.

An interesting example of the consequences of photo-induced oxidation is given by the formation of lipid hydroperoxides. These molecules, which can be formed either by singlet oxygen or radicals, are more polar than their non-oxidized counterparts. In terms of photooxidation reactions in the presence of oxygen, the most probable start is the reaction of oxygen with the triplet excited state, forming singlet oxygen. This reaction usually has diffusion-limited velocity, compared with the direct reaction of the triplet with a double bond in a long chain substrate, whose bimolecular rate constants are in the order of 10^4^ L·mol^−1^·s^−1^ or smaller (for polyunsaturated lipids, this value increases significantly) [26]. When organized in a lipid bilayer, the –OOH group is stabilized by interaction with the polar-heads region of the membrane. This introduces a bend in the fatty acid chain, increasing the area occupied per lipid. Therefore, the lipid bilayer increases in area and decreases in thickness [54,55]. This conformational change can also account to phase separation of initially homogeneous membranes when exposed to PS and light. Importantly, changes in the domain organization of membranes is known to affect cell signaling, and probably pathways related to apoptotic cell death [35,44,56,57,58]. Considering that one of the main events of apoptosis is the detachment of cytochrome *c* from mitochondria, Kawai *et al.* demonstrated that lipid oxidation and reorganization within the membrane change the binding of cytochrome *c* to liposomes that mimic the inner mitochondrial membrane (IMM). That is, in the presence of 1-palmitoyl-2-azelaoyl-*sn*-glycero-3-phosphocholine (PazePC), which bears a nonreactive carboxylic group, the dissociation constant between cytochrome *c* and IMM was increased, while in the presence of a mixture of two hydroperoxide isomers derived from 1-palmitoyl-2-oleoyl-*sn*-glycero-3-phosphocholine (POPC), which have a reactive peroxide group, this same dissociation constant was lowered [59].

However, even for lipid oxidation, some effects still cannot be easily understood. For example, membrane permeabilization and/or lysis are known consequences of lipid photooxidation. If the contents of the cell leak, the consequences may be lethal [60,61,62]. The identity of the products leading to increased permeabilization is not well characterized yet. Up to now, there is evidence that hydroperoxides are capable of maintaining chemical gradients across the membranes, and that truncated oxidized lipids (aldehydes and carboxylic acids) should play a major role [55,63,64,65,66]. The formation of this truncated lipids depend not only on the formation of singlet oxygen, but instead on the direct reaction of the triplet with either the hydroperoxide or the double bond. However, the detailed pathways by which the initial lipid oxidation leads to membrane leakage are still under debate. For other kinds of biomolecules, structure-activity relations are even less clear.

Although photooxidation of amino acids can bring a lot more different structural changes to proteins, compared with the relatively simple conformational changes expected for lipids, it is well known that it has consequences in activity, mechanical properties, aggregation state, and affinities to ligands. Indeed, photooxidation of enzymes can lead to loss of activity, which can be further decreased in subsequent chemical dark steps [17,18,67,68,69,70]. Oxidation of a first protein or peptide can also lead to oxidation of other proteins, during the propagation of the radical chain reaction or by further reactions between oxidized molecules. Interestingly, inactivation of caspases (which are thiol-dependent cysteine proteases) in the presence of peroxide-containing peptides generated by photooxidation is reported, indicating that protein oxidation can directly affect cell death mechanisms [67].

When anti-apoptotic proteins such as B-cell lymphoma 2 (Bcl-2) and B-cell lymphoma-extra large (Bcl-x_L_), for example, are photodamaged, their function of preventing the release of mitochondrial apoptogenic factors such as cytochrome *c* and AIF (apoptosis-inducing factor) into the cytoplasm is compromised. The release of this apoptogenic factors into the cytoplasm activates caspases, which cleave a set of cellular proteins promoting apoptotic pathways [71,72]. Kessel *et al.* reported that high-dose PDT with 9-capronyloxytetrakis-(methoxyethyl)porphycene (CPO) resulted in a substantial loss of Bcl-2, initiating the intrinsic apoptotic pathway [73]. Also, inhibition of mammalian target of rapamycin (mTOR) complex (a serine/threonine protein kinase that regulates cell growth and belongs to the phosphatidylinositol 3-kinase related kinase protein family) by photodamage can induce autophagy, resulting in autophagic cell death [74,75]. Weyergang *et al.* demonstrated that *cis*-disulfonated aluminum phthalocyanine (AlPcS_2a_) targets photodamage in mTOR signaling network, resulting in attenuation of mTOR signaling as an important mechanism for AlPcS_2a_-PDT mediated cell death [75]. In contrast, key autophagic proteins such as Beclin 1, autophagy-related protein 5 (Atg5) and autophagy-related protein 7 (Atg7) appear to be unaffected in PDT protocols [74,76].

It is also possible to seek for the effects of photooxidations at organelle level and progress towards cellular outcomes. Since Golgi apparatus and endoplasmic reticulum (ER) are associated to protein synthesis and processing, one could expect that photodamage to these organelles could significantly compromise the production of these biomolecules [77,78]. ER is also the major reservoir of intracellular calcium [79]. In principle, damaging this organelle can release a burst of calcium that can deregulate several signaling pathways and even trigger cell death mechanisms [80]. Intracellular calcium burst has been shown to play a major role in inducing programmed necrotic cell death [81], as well as to affect negatively the ER homeostasis with mitochondria [82]. However, the specific role of calcium as the responsible agent for cell death during/after PDT has been controversial, perhaps because in PDT several molecular targets are damaged at the same time. The potent inhibitory effect of Cyclosporine A on the start of the mitochondrial permeability transition pore, which is induced by Ca^2+^, suggested an important role for calcium in causing PDT-related cell death [83]. PDT with aluminum phthalocyanine (AlPc) caused an expressive increase in intracellular calcium concentration. However pre-treatment of cells with calcium chelators, caused enhancement instead of decrease of cell death, putting doubt on the active role of calcium as the cell-killing agent [84]. Buytaert *et al.* reported that photodamage to ER leads to an immediate loss of sarco/endoplasmic reticulum Ca^2+^-ATPase (SERCA2) protein levels, causing disruption of Ca^2+^ homeostasis and cell death after hypericin-mediated PDT [85]. However, a more recent study on PDT targeting ER, suggested that SERCA2 is not the main target, but calcium dependent cysteine proteases (calpains) activation, which is the main responsible for programed necrotic cell death. These results show that photodamaging ER and consequently the release of a burst of calcium in the cytosol clearly affects cell viability. However, the detailed mechanistic consequences of this burst needs further investigation. Additionally to ER, targeting mitochondria and lysosomes also has an important role in cell death mechanism.

It is well-defined in the literature that mitochondria are key organelles to apoptotic mechanisms, since photodamaging them allows the release of cytochrome *c* to the cytoplasm. This activates caspase cascades (similar consequences of photodamaging Bcl-2) and results in apoptosis. However, it is noteworthy that mitochondrial photodamage can provide not only apoptotic cell death, but also trigger necrosis or autophagy, depending on the PDT-dose (which depends on PS concentration and light intensity). High PDT-dose levels causes drastic mitochondrial permeability and ATP levels depletion, leading to necrosis. Mild PDT-dose levels triggers apoptosis (as already mentioned), and low PDT-dose levels promotes limited mitochondrial permeability and induces autophagy (“mitophagy”). In this case, autophagy happens to protect cells by recycling damaged mitochondria as a repair mechanism [86,87,88]. If this protection mechanism of autophagy fails, autophagic cell death can be triggered [89,90,91].

Targeting lysosomes also provides necrosis, apoptosis, and autophagic cell death mechanisms, depending on the PDT-dose. High doses of PDT induce complete breakdown of this organelle releasing high concentrations of lysosomal enzymes (such as cathepsins B, D, L) into the cytoplasm, resulting in unregulated necrosis [92,93]. On the other hand, partial damage to lysosomes causes the release of hydrolases, which are able to cleave pro-apoptotic proteins, triggering apoptotic cell death and/or inhibition of the autophagic flux. This latter phenomenon compromises cytoprotection mechanisms and leads to autophagic cell death [89].

Although the most potent PSes are usually localized in mitochondria and/or lysosomes and their accumulation in the nucleus is not so common, DNA may also be oxidized in PDT treatments. Porphyrins and/or metalloporphyrins can cleave nucleic acids via oxidative attack on the sugar moiety or by photo-induced mechanisms involving singlet oxygen, leading to single strand breaks on plasmid DNA and directly oxidizing cellular DNA [20,94,95,96]. However, DNA photodamage has not been directly linked to lethal effects in PDT [95,96]. Furthermore, oxidative DNA damage has mutagenic potential, since it has been suggested that radical reactions may be involved at several points in the multistep process of chemically-induced carcinogenesis [95,96]. This shows that, in the same way as we should look for desirable targets for PDT, it is also necessary to understand which reactions should be avoided in order to control possible side effects and cell death mechanisms.

**Figure 2:**
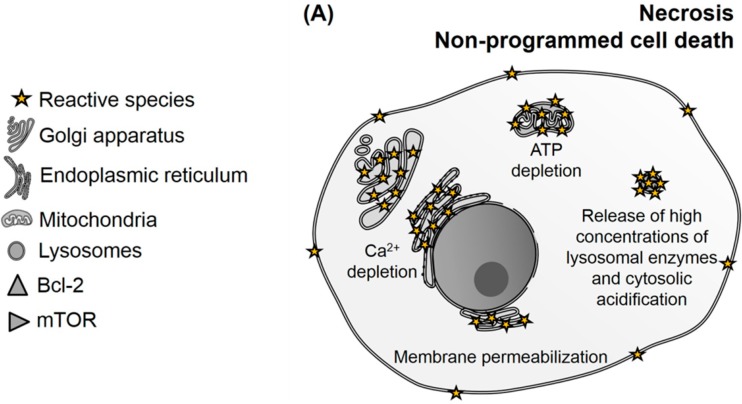

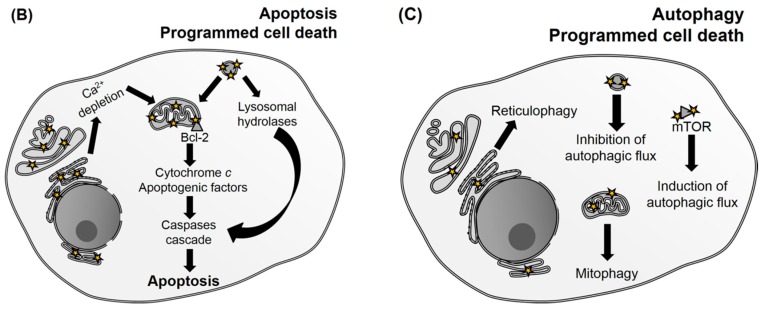
Biological outcomes of photodynamic therapy (PDT) associated with the amount of reactive species that are generated and possible targets of photooxidation. Note that it is not necessary to damage all targets together to trigger cell death. (**A**) High PDT-doses in organelles and photodamage of plasma membrane cause ATP depletion, resulting in non-programmed cell death (necrosis); (**B**) production of reactive species in mitochondria and/or damage to protein B-cell lymphoma 2 (Bcl-2) causes the release of cytochrome *c* and other apoptogenic factors, which classically trigger the caspases cascade, resulting in apoptosis. Photodamaging endoplasmic reticulum (ER) and lysosomes can also converge to mitochondrial damage, resulting in apoptosis; (**C**) low PDT-doses in organelles (mitochondria, ER and lysosomes) may activate autophagic process in an unbalanced manner (too much induction or flux inhibition), resulting in autophagic cell death. The inhibition of the mammalian target of rapamycin (mTOR) complex by photooxidation can also trigger autophagy as a cell death pathway.

Identifying correlations between chemical targets and cell death pathways (Figure 2), as well as their advantages and disadvantages, is desirable. For many years, apoptosis was considered the most wanted mechanism of programed cell death in PDT, due to its lack of side effects if compared to necrosis. However, it is know that cancer cells can be resistant to apoptosis [97]. Consequently, autophagic cell death has been emerged as alternative to provide more efficient PDT treatments to cells deficient in apoptosis. In general, high PDT-doses and/or uncontrolled photodamage lead to uncontrolled release of biomolecules from non-programed cell death into the extracellular space, initiating an inflammatory response in the surrounding tissue. For this reason, necrosis is generally seen as an undesirable mechanism. On the other hand, a specific photodamage in suitable PDT-doses can be lethal to cells without injuring the surrounding healthy cells. This explains how important it is understand which targets and PDT-doses are suitable to reach better efficiency with minimal side effects. Table 1 provides a list of some selected PS, together with their subcellular localization, biological consequences and induced mechanisms of cell death.

**Table 1:** Subcellular localization, biological consequences and cell death mechanisms induced by photosensitizers (PSes).

Photosensitizer (PS)	Subcellular Localization	Biological Consequences	Cell Death Mechanism	References
9-Capronyloxytetrakis-(methoxyethyl)porphycene (CPO)	Endoplasmic reticulum (ER)	B-cell lymphoma 2 (Bcl-2) loss and release of Ca^2+^	Apoptosis and autophagy	[73,98]
Sulfonated aluminum phthalocyanines (AlPcS_2-4_)	Lysosomes	Photodamage to mammalian target of rapamycin (mTOR) signaling network and release of lysosomal proteases, which activate caspase 3	ND	[75,99]
Benzoporphyrin (BPD, Verteporfin)	Mitochondria	Decreases B-cell lymphoma-extra large (Bcl-x_L_) and increases the Bcl-2 associated X protein (Bax)/Bcl-x_L_ ratio	Apoptosis	[100,101]
Cationic porphyrins	Plasma membrane and mitochondria	Plasma membrane disruption and mitochondrial inner membrane permeabilization, causing release of cytochrome *c*	Necrosis and apoptosis	[102,103,104]
Cationic zinc(II) phthalocyanines	Mitochondria	Destruction of the inner mitochondrial membrane	Apoptosis	[105]
Chlorophyllin e4	Mitochondria and lysosomes	ND	Apoptosis and autophagy	[88,106]
Hypericin	ER	Loss of SERCA (sarco/endoplasmic reticulum Ca^2+^-ATPase) protein levels causing ER-Ca^2+^ depletion	Apoptosis and autophagy	[85,107,108]
Methylene blue (MB)	Mitochondria and lysosomes	Reduction of mitochondrial membrane potential and downregulation of the anti-apoptotic proteins Bcl-2	Apoptosis	[31,42,109]
mTHPC, Foscan^®^	Mitochondria, golgi apparatus and ER	Photodamage to Bcl-2 protein and release of cytochrome *c*	Apoptosis	[110,111,112]
*N*-Aspartyl chlorin e6 (NPe6)	Lysosomes	Release of lysosomal proteases that cleave BH3-interacting domain death agonist (Bid)	Apoptosis	[113]
Photofrin^®^	Plasma membrane and mitochondria	Plasma membrane disruption and mitochondrial inner membrane permeabilization, causing release of cytochrome *c*	Necrosis and apoptosis	[114,115,116,117]
Rose bengal (RB)	Golgi apparatus	ND	Necrosis, apoptosis and autophagy	[118,119,120,121]
Silicon phthalocyanine (Pc4)	Mitochondria, ER and Golgi	Photodamage to Bcl-2 protein	Apoptosis	[122,123,124]
Tetrakis (p-sulfonatophenyl) porphyrin (TPPS_4_)	Lysosomes	Release of proteases causing cathepsin-mediated cleavage of Bid and inhibition of autolysosome formation	Apoptosis and autophagy	[72,125,126,127]

ND: Not determined.

Aside from the amount of damage, another property that may affect the outcome of PDT is the intrinsic photochemical properties of the PS. Few reports distinguish between the consequences of singlet oxygen or radicals in photosensitized cells. It is important to mention the work of Kochevar that showed that pure singlet oxygen generation by visible light irradiation of rose bengal (RB) causes mainly apoptosis, while UVA irradiation of a derivative causes formation of both singlet oxygen and radicals, damaging the plasma membrane and leading to necrosis [128].

It is possible to draw some conclusions from this analysis to photooxidations in PDT: (i) the diffusion of photoinduced reactive species in biological environment is severely limited and therefore, photodamage occurs in the nanoenvironment where the photon is absorbed; (ii) the majority of molecular oxidation starts with singlet oxygen, but radical-mediated reactions are fundamental to form important intermediate species, which allow to achieve points of no-return cellular damage; (iii) targeting specific biomolecules can trigger different mechanisms of programmed cell death; (iv) oxidation of specific targets may lead to a better PDT effect than generic and extensive oxidation.

## 3. Parameters Determining Photosensitizer (PS) Efficiency

### 3.1. Biological Environment Affects Triplet Reactivity

**Figure 3:**
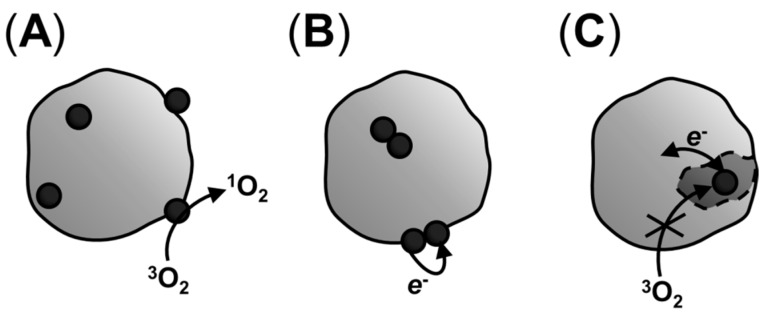
Possible outcomes of the interaction between photosensitizers (PSes) and binding sites of macromolecules or particles, considering triplet excited states deactivation pathways: (**A**) stabilization of the monomeric form of the PS and formation of singlet oxygen by energy transfer; (**B**) stabilization of the dimeric species, favoring the dye–dye mechanism; and (**C**) binding of the PS to a pocket inaccessible to oxygen, raising the probability of electron transfer pathways.

One important topic in PDT research is the quest for PSes with enhanced activity. A common practice is to measure singlet oxygen generation quantum yield (Φ_Δ_) in isotropic solution and, as singlet oxygen is usually considered the main species taking part in PDT, the PSes with higher Φ_Δ_ are usually regarded as the more promising ones [1]. However, there are many pitfalls in this strategy, and many studies show that Φ_Δ_ does not always correlate with photodynamic efficiency [42,47,103,129,130,131,132]. This happens because the properties of the PS (ground and excited states) can be affected by interaction with the biological environment, and also because cells are complex heterogeneous systems and the spatial distribution of PSes defines where the oxidizing species will be generated (Figure 3).

Regarding this latter factor, one of the simplest effects to be understood is the fact that among PSes with similar Φ_Δ_, the ones that interact more with biomolecules (such as lipids packed into bilayers) lead to more extensive photodynamic damage. Considering the small diffusion distance of singlet oxygen (as well as of other reactive species), it is not surprising that proximity to biological targets should affect the extent of photooxidation [89,103,111,130,133,134,135,136,137,138]. Another effect that arises from the localized generation of reactive species is the variation of their lifetimes in different subcellular localizations and also if compared to isotropic solution [42,139]. Oliveira *et al.* incubated HeLa cells with methylene blue (MB) or crystal violet (CV) in the presence of deuterium oxide and showed that, while the former led to a lifetime of 33 μs, the latter led to 5 μs. Both lifetimes were smaller than expected for pure deuterium oxide, suggesting that the PSes experiment environments (*i.e.*, subcellular localizations) with different capabilities to quench singlet oxygen. However, the singlet oxygen molecules generated by CV were a lot more efficient in causing cell death, and this correlates with its smaller lifetime [42]. Therefore, differences in diffusion distance should be expected depending on the site of generation of singlet oxygen, affecting the probability that it reacts with the desired targets especially when physical quenching is taking place.

Triplet excited state deactivation pathways (*i.e.*, electron or energy transfer) and, hence, the efficiency of singlet oxygen generation, will also be changed in the biological medium if compared with isotropic solutions. Many PSes can act both by electron or energy transfer, provided that the triplet excited state has enough energy to sensitize singlet oxygen formation and redox potentials compatible with existing substrates [34,40,140]. The relative occurrence of each process will depend on factors such as their rate constants, the concentration of oxygen or other substrates in the surrounding medium and also on interactions with other molecules or with PS molecules on their own [1,3,16,141,142,143,144]. All these factors will depend on the nanoenvironment where the PS is: lipid membranes, for example, are richer in oxygen than the surrounding solution, and PSes that localize deeper on the membrane encounter higher concentrations of oxygen [50]. Ding *et al.*, for example, showed that 5,10,15,20-tetrakis(meso-hydroxyphenyl)porphyrin (mTHPP) encapsulated in polymeric micelles results in greater PDT photototoxicity against cancer cells under hypoxic conditions due to significant increase in generation of a superoxide radical in the electron-rich micelle core, competing with singlet oxygen generation. Indeed, electron transfer becomes dominant under hypoxic conditions [129]. The pathway followed by the triplet excited state may also change during the course of irradiation, due to PS chemical and photochemical transformation, as well as availability of novel substrates (photooxidized molecules), the presence of highly prevalent reducing species and/or depletion of initial reactants (such as oxygen or radical reaction substrates).

The role of PS aggregation on its photochemical and photophysical pathways is studied for several classes of PSes and in many different systems. Aggregation, which in most cases can be simply identified by changes in electronic spectra of the PS, can happen as a result of high PS concentration and also be affected by ionic strength, temperature and interaction with molecules that stabilize differently the monomeric or the aggregated form of the PS [141,142,145,146,147,148,149,150]. In some cases, aggregation just diminishes the activity of the PS, whereas in others it may change its mechanisms of action. The cationic PS MB, for example, aggregates in the presence of negatively charged interfaces, such as sodium dodecyl sulfate (SDS) aggregates. While in high MB/SDS ratios MB dimerizes, the equilibrium is shifted towards the monomeric form of the PS when this proportion is lowered. Interestingly, laser flash photolysis measurements show that the lifetime of the triplet excited state increases from 40 ns to 1.5 μs upon raising SDS concentration. Whereas the latter lifetime corresponds to quenching by oxygen forming singlet oxygen, the former is ascribed to a dye–dye electron transfer, resulting in MB-derived radicals [141,142]. This phenomenon is expected to affect the intracellular PDT efficiency of MB, given that it can lead to radical reactions. It turns out that MB’s aggregation equilibrium is also affected by binding to mitochondria, being dependent both on mitochondrion membrane potential and on the relative concentration between MB and these organelles [151].

Indeed binding to biomolecules is known to alter PS photochemical and photophysical pathways, whether or not by affecting aggregation equilibriums. Depending on the polarity of the PS and the composition of lipids (and hence the properties of the lipid bilayer), lipid membranes can stabilize either the monomeric or the aggregated forms of the PS, thus affecting the generation of reactive species [130,142]. For PSes that aggregate in aqueous medium and have similar Φ_Δ_ in solvents that prevent aggregation, binding to lecithin liposomes led to higher singlet oxygen generation than major partition in the surrounding solution [130]. On the other hand, an increase in electron transfer processes may also be expected, since binding to membranes increases the concentration of possible substrates to these reactions.

PSes can also bind to proteins, both to specific binding sites or in an unspecific manner, altering their photosensitizing properties. Of course, the observed effects will vary with the identity of the protein, PS and with their relative concentrations. When RB binds to bovine serum albumin (BSA) in a non-specific manner, RB cannot sensitize singlet oxygen formation as a result of static quenching of its excited state due to aggregation. On the other hand, in lower concentrations RB binds to the hydrophobic pocket of the protein, still generating singlet oxygen. However, under these conditions singlet oxygen can be quenched by BSA itself [152]. When PS-protein complex are excited, direct substrate-PS reactions are favored leading to a decrease in the yield of singlet oxygen and usually to the formation of adducts [153]. Besides interfering in aggregation equilibriums, proteins can also decrease nonradioative relaxation of PSes, with subsequent enhancement of the photoreactivity of the dye, as observed with triarylmethane dyes in the presence of BSA [153].

Therefore, it is clear that PS photochemistry and photophysics are dependent on the medium where the PS is through a complex set of interactions. This knowledge can, in principle, be used to increase photodynamic efficiency, by designing formulations that regulate PS photochemistry and photophysics. Modulating PS aggregation equilibria by controlling the ionic strength or stabilizing the most active species of the PS are possible strategies [147,154,155,156]. In view of that, Nuñez *et al.* recently reported that urea stabilizes the monomeric MB species, leading to a higher efficiency of antimicrobial PDT against *Candida albicans* [147]. Other strategies to control aggregation consist in the use of nanoparticles with specific rates of dimers and monomers [157,158,159,160,161], binding to biomolecules [162] or to synthesize molecules with groups that hinder aggregation [163,164,165]. For example, Tada *et al.* compared three different types of silica nanoparticles containing thionin or MB at different ratios of dimer to monomer and showed that nanoparticles with lower PS dimer/monomer ratio presented higher generation of singlet oxygen [160].

Direct studies of PS photophysics and photochemistry in cells are still scarce. Fluorescence lifetime imaging microscopy (FLIM) has been used to assess variations in the fluorescence lifetime of PSes due to subcellular localization. By measuring the fluorescence lifetime of Photofrin^®^ in MatLyLu (MLL) cell line, Yeh and coworkers showed that, depending on the PS incubation time, subcellular localization changed [114]. Other works have also reported that PS fluorescence lifetime is sensitive to intermolecular interactions and changes in nanoenvironment due to the different subcellular localization [166,167,168,169]. These results demonstrate that the singlet excited state of PSes is affected by interaction with intracellular environment. Up to now, there are no straightforward methodologies to study triplet excited states in biological samples. Nonetheless, it is expected that changes in triplet excited state generation quantum yield and lifetime should also happen under these conditions.

An important take-home message is that transposing data obtained in isotropic solutions (e.g., Φ_Δ_) to biological conditions may lead to pitfalls and incorrect choices of PSes. Therefore, careful studies should be done in conditions as close as possible to the real biological scenario. Pursuing this challenging endeavor is worthy, since revealing the specificities of each situation may be the key to better and target-based PDT protocols.

### 3.2. The Biological Outcome as a Function of PS Properties

**Figure 4:**
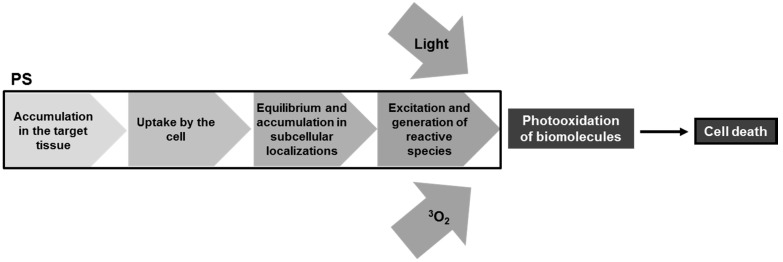
Proposed main steps that should happen with the photosensitizer (PS) in order to achieve cell death.

When cells are treated with PS, a number of steps must take place before cell death is achieved. Namely, the PS must be internalized by the cell, equilibrate and accumulate in subcellular localizations, be excited and only then oxidize biomolecules. At the same time, light must reach the absorbing molecules, as well as oxygen should be available or properly delivered (Figure 4).

All of these steps turn out to be decisive to the final PDT efficiency. However, predicting them all is a mighty task. Take the case of tumors, for example, some PSes compromise tumor cells mainly in an indirect way, damaging the tumor vasculature and blocking the supply of molecular oxygen and essential nutrients. This effect is associated to binding of the hydrophilic/water soluble PSes to serum albumin, since it mediates PS accumulation in vascular stroma, or to binding to vascular structures/constituents, such as collagen [170,171,172]. On the other hand, there is evidence that highly hydrophobic PSes act in tumors mostly by direct interactions, since they are usually transported inside the body by association to low density lipoproteins (LDL), which can deliver them to intracellular sites [172,173,174]. However, studies in tumor level are very far from the molecular scale, and this kind of analysis is rather complex. For that reason, many PDT protocols are empirical and do not reach the maximum efficiency. In this section, we discuss how to enhance the efficiency of PDT based on biological considerations at molecular and cellular levels, trying to relate them as much as possible to the chemical background discussed above.

One important issue concerning the specificity of the photooxidative damage is the selective localization of the PS in the target tissue if compared to normal cells. Most of the commonly used PSes present low selectivity for the tumor tissue, typically achieving ratios of 2–5:1 in tumors *vs.* normal tissues, resulting in phototoxic side effects [175]. Moreover, although many different tissues can retain the PS after its administration to the patient, their elimination rates can be different. Therefore, it is important to carefully choose a time delay between the administration of the drug and the irradiation procedure, so that the PS concentration ratio between tumor and normal tissue reaches a maximum [176].

In order to increase selectivity, targetable and activatable PSes or nanocarriers have been designed, exploiting specific biochemical features of the tumors. Usually tumors present higher glycolysis rates, higher serum albumin turnover, and overexpresses LDL and epidermal growth factor (EGF) receptors in comparison to healthy cells. For that reason, PSes have been coupled to molecules like sugars [177,178,179], serum albumins [180,181], LDL [182,183], and EGF [184,185]. Some specific biomarkers overexpressed in tumor cells have also been used to further concentrate PS in tumors. In addition, PSes conjugated to antibodies, peptides ligands, and proteins exhibits special targeting, as well as PSes conjugated to non-protein ligands (e.g., folic acid) have been proposed [180,186,187,188,189,190]. Another strategy is to use pH-activatable PSes, which respond to the higher acidity of cancer cells, and glutathione-activated PS, since glutathione concentration is also higher in these cells [191,192,193,194]. More than one of these strategies can be used at the same time, resulting in enhanced selectivity [195]. Another strategy that has called a lot of recent attention is the use of inorganic complexes, in which each of the coordination sites to the central inorganic metal can carry organic molecules that execute different actions [196].

In addition to the above-discussed strategies to improve PS delivery to its target, approaches have also been developed to overcome low oxygen concentrations or difficulties to shed light on the PS. Excitation of the PS requires irradiation with a suitable light source, which matches the maximum absorption wavelength of the PS. In melanoma cells, melanin absorbs a significant amount light in the visible region of the spectrum, competing with the PS towards light absorption and decreasing the efficiency of PDT. A recently-developed tactic to overcome this difficulty is the use of upconversion PSes/nanomaterials, since they convert photons with energy corresponding to the near-infrared spectral region (which are not so efficiently absorbed by melanin) to a higher-energy output photon. Therefore, the shorter-wavelength irradiation is generated *in situ*, leading to higher probability of PS excitation [197,198,199].

A striking difficulty faced in the treatments of solid tumors by PDT is the low oxygen supply in some areas of the tumor mass (hypoxia). One of the proposed workarounds for this problem is to develop formulations that can locally produce oxygen. An interesting example is the highly selective and efficient MB-based nanoparticle developed by Chen *et al.*, which is α_V_β_3_ integrin-targeted and hydrogen peroxide-activatable, being able to evolve oxygen in hypoxic tumors [200]. Some attempts have been made in order to use inorganic salts to improve PDT efficiency, since they are able to generate oxygen-independent radicals. Recently, it was also demonstrated that azide acts by an oxygen-independent mechanism [22,201,202,203]. The use of iodide and bromide also showed to be effective to enhance PDT inactivation of bacteria, and the mechanism seems to be oxygen independent [204,205].

Once the target cells are reached, the mechanisms of cellular uptake can vary according to the PS. There are three important properties that control both PS uptake, and subcellular localization: the degree of lipophilicity, the type and number of charges and the degree of asymmetry present in the molecular structure [103,156,206]. Most PSes consist in a chromophore with attached side groups. As expected, many works show that the presence of lipophilic side chains around the chromophore unit increases PS lipophilicity [48,103,156]. The degree of lipophilicity of a PS can be related to its log *P* value (logarithm of the *n*-octanol/water partition coefficient) and this parameter has been often used to predict the relative tendency of the PSes to interact/bind to biological membranes. Usually, the higher the log *P* value, the higher the interaction with membranes. However, relying solely in log *P* values to predict PDT outcome or even to predict membrane binding, can lead to pitfalls. The interaction with lipid membranes cannot be always predicted by the log *P* value, for the asymmetry of PS side groups and charges can maximize intermolecular interactions with lipids in membranes. Increasing the length of the alkyl chains above certain limits leads to aggregation and suppression of cellular uptake, decreasing the PDT efficiency of the PS [48,156]. In addition, cationic amphiphilic porphyrins with two adjacent positive charges (*cis* isomer) presents higher uptake and photodynamic efficiency than cationic porphyrins with two opposite positive charges (*trans* isomer), since the former have a structure which allows a deep penetration in lipid membranes [133].

In terms of cellular uptake, internalization occurs by diffusion in few cases, mostly happening via endocytosis or membrane pumps. Relatively hydrophilic PSes, bearing polar or charged side chains, are too polar to cross biological membranes by diffusion, being usually internalized by endocytosis [207,208]. However, some PSes with up to two charges can still diffuse across the plasma membrane, provided they are sufficiently hydrophobic [209]. Furthermore, among the PSes that are internalized by endocytosis, the series of events taking place can classify the mechanisms as caveolae or clathrin-dependent uptake, clathrin-independent, *etc.* [210,211]. Unfortunately, little information is available about how the mechanisms of PS cell internalization affect the extension of its uptake and also its subcellular localization [211,212].

Perhaps the most important parameter in terms of the PDT outcome is PS subcellular localization. Remember that usually the site of generation of reactive species is also their site of action [39,42]. An important parameter to predict subcellular localization is the charge of the PS. For instance, positively charged porphyrins, phenothiazines, tryarylmethanes, rhodamines, and cyanines localize mainly in mitochondria since they are electrostatically attracted by its negative electrochemical transmembrane potential, being up to 100-times more concentrated than in the cytoplasm [47,176,213,214,215,216]. Oppositely, anionic PSes as mono-l-aspartyl chlorin e6, meso-tetra-(*p*-sulphophenyl)porphine, and disulfonated aluminum phthalocyanine (AlPcS_2a_) tend to localize in lysosomes after their cellular uptake by endocytosis [113,127,217,218,219,220]. PSes that are taken up by endocytosis may localize in lysosomes because endosomes follow the intracellular trafficking and end up fusing with lysosomes. Additionally, dyes that bear weak base amines can accumulate in these organelles. This happens because they enter lysosomes in their uncharged form, but become trapped once protonated due to the low pH inside this organelle [221,222,223].

The symmetry of charge distribution can also affect the organelle in which the PS will accumulate. Kessel *et al.* studied two meso-tetraphenylporphyrin derivatives bearing two cationic trimethylamonium groups in adjacent and opposite positions. Whereas the asymmetrical cationic compound penetrates the plasma membrane by diffusion and targets the mitochondria of murine leukemia cells, the symmetrical cationic compound localizes in the lysosomes, probably by an endocytic uptake mechanism [224]. PS subcellular localization may also change during the PDT treatment. For example, usually lysosomal damage results in leakage of its content and PS spreading to cytoplasm, promoting its reallocation [225,226,227,228,229,230,231].

Being the PS accumulated in its target organelle(s), it should produce at least some amount of reactive species in order to properly perform photooxidation of biomolecules. Therefore, decreasing the tendency to photobleaching, aggregation and reduction by the intracellular environment will be important. Physicochemical parameters that could be used to evaluate these properties are photobleaching rates, PS reduction potential, and aggregation constants [96,147,232,233,234]. For example, MB gets reduced inside mitochondria, generating a semi-reduced radical after the first one-electron reduction and yielding leuco-MB after a second reduction. Given that the mitochondrion is one of the possible subcellular sites of MB accumulation and that neither the semi-reduced radical nor its leuco form absorb light in the visible, they no longer act as PSes, decreasing therefore the PDT efficiency [42,151]. Methylated derivatives of MB, such as 1-methyl MB and 1,9-dimethyl MB, besides presenting slightly higher Φ_Δ_ and being more hydrophobic, are more resistant to reduction and also more phototoxic to EMT-6 cell line [235].

Given that low dark toxicities are also needed in PDT protocols, strategies that further increase the efficiency of the treatment and raise the light to dark ratio of toxicities should be pursued. The combination of PS with other drugs—or even other PSes [236]—has been exploited to enhance PDT efficiency. A recently-developed approach is designing new PSes with dual action mechanisms. Albani *et al.* synthesized a ruthenium complex that not only efficiently generates singlet oxygen, but also simultaneously releases drugs during light activation [196]. A similar strategy, using a combination of a porphyrin or phthalocyanin PS and a platinum complex, was also reported [237,238]. Another possibility to enhance the PDT effect in tumors is using it as tool to deliver chemotherapeutic drugs taken up by endocytosis in a method called photochemical internalization (PCI). The principle of PCI protocols is to address the PS to the endocytic vesicle membrane and then use photoinduced damage generated by a PS to release the drug. The main advantage is that, without the action of the PS, the drug could be degraded in lysosomes and have its activity reduced [239,240].

It is important to keep in mind that, even though efforts have been made to find relationships between the chemical structure of the PS and biological consequences, we are far from a crystal-clear picture. This happens due to the difficulty to relate information from more simple experiments to complex biological systems as eukaryotic cells and tissues. For now, we can point out two main steps for planning to target based PDT: (i) choose a target (or a set of targets) whose damage leads to a specific and desired kind of cell death; (ii) choose a PS that targets the subcellular localization of this target and study if it is able to oxidize the chosen target. Here it should be considered how PS photochemistry and photophysics may work under these conditions and also if the PS has suitable characteristics for PDT, such as absorbing light in the so called therapeutic window.

## 4. Major Challenges

In the previous sections of this review, we presented an overview on the mechanisms taking place in PDT, discussed possible targets of photooxidative damage, ways to aim for them and finally potential manners to maximize PDT efficiency. The final aim would be, of course, to target specifically-diseased tissues and, inside these tissues, induce specific mechanisms of cell death by light. This would allow cells to “melt down” or kill themself with the minimum amount of damage in surrounding tissues. Here we discuss some of the major challenges still remaining on this domain and suggest some strategies to surpass them.

Nowadays, the enhancement of PS efficiency is mainly done by optimizing properties like Φ_Δ_ and light absorption in the therapeutic window, modulating PS hydrophobicity, and avoiding aggregation and photobleaching. Although many different structures of PSes have been proposed and many of them present improvements if compared to preceding ones, none of them seem to be close to a magic bullet and no paradigm shift was achieved in the last decades. Nonetheless, we believe that a breakthrough can arise by seeking target-based PDT. A careful planning of targets and PSes suited for each condition should allow very efficient treatments. In this case, PDT protocols should be sought in a tailor-made way and the desired photooxidative outcome should be used in order to choose the PS. Depending on the tumor, for example, different targeting strategies could be used. As an example, the development of PS-immunoconjugates allows that monoclonal antibodies linked to the PS specifically recognize molecular markers present in the surface of some tumor cells (e.g., EGF receptor, which is mainly overexpressed by cancer cells). Savellano *et al.* demonstrated that the PS verteporfin conjugated to a monoclonal antibody that target EGF receptor led to a reduction of 90% in the viability of ovarian cancer metastasis, while no significant effect was observed in EGF receptor-negative cells [241]. An important thing to remark is the outline proposed in Figure 4. We believe that generation of reactive species is only one step among the many steps that matter to succeed in PDT, and that optimum spatial distribution is a key factor to achieve the desired biological consequences.

Most cell-based studies do not consider the dynamics happening inside the cell, nor its molecular organization. Very few studies pinpoint which are the surfaces to which the PS binds inside a certain organelle or define if it remains freely-diffusing in the aqueous compartment of this organelle. This is important because, in order to selectively activate certain cell death pathways, not only must the PS be in the correct organelle, but also be close enough to the chemical targets. Although many co-localization studies allow the assignment of the localization of a certain PS to a specific organelle, these studies do not consider that there are binding and dissociation equilibria taking place, and that PS can localize in different places to different extents (*i.e.*, quantitative data still lacks). Indeed, it is improbable that a PS binds only to one kind of biomolecule, possibility interacting with many different ones. In this sense, we believe that ranking key targets to promote apoptotic (e.g., Bcl-2) and autophagic cell death (e.g., mTOR) should be a priority in PDT research. Of course, many other leading targets are still to be discovered, and studying the effects of oxidized biomolecules (not only proteins) in cells and accessing structure-activity relationships should be a priority.

Another difficulty still faced by scientists working with PDT is the assignment of the photophysical and photochemical mechanisms taking place in complex scenarios (e.g., cells), as well as characterization of the generation and deactivation of reactive species in these same environments. Singlet oxygen imaging remains a true challenge, for its faint emission at 1270 nm and the low efficiency of detectors in this spectral range make difficult the acquisition of high-contrast images. The assignment of the distribution of radicals in cells is also not routine yet, although many specific probes are being developed to indirectly achieve spatial resolution. This is the case of boron-dipyrromethene–α-tocopherol probes that are being used to detect and image peroxyl radicals [242,243].

In addition to generation of reactive species, it is also difficult to identify reaction products in living cells. The products of oxidation can be very diverse, and “omics” (*i.e.*, lipidomics, proteomics *etc.*) approaches may be needed in order to identify a complete set of oxidized lipids or proteins. Some studies have identified hundreds of proteins which have been oxidized during PDT, resulting in thiol oxidation and carbonylation. Among them, it is worthy of mention metabolic enzymes, proteins that facilitate protein degradation, chaperones, structural proteins and the enzymes related to energy metabolism [29,30,31]. On the other hand, characterization of the oxidized lipids generated by PDT is still poor and there are only few studies. Alves and coworkers, for example, showed that oxidation of phosphatidylethanolamines is present in *E. coli* membranes after PDT with (5,10,15-tris(1-methylpyridinium-4-yl)-20-(pentafluorophenyl)porphyrin triiodide as PS [32]. However, there is still lack of methodologies on this field and also of extensive databases to deal with oxidized molecules. From the point of view of molecular biology, the transformations suffered by the cell due to photoinduced damage are still not yet fully characterized. Liu *et al.* identified three different genes (matrix metalloproteinase 10 (*MMP10*), Ras homolog enriched in brain like 1 (*RHEBL1*) and LDL receptor (*LDLR*)) whose expression increased due to PDT with four different PS that have different subcellular localization. Although the pathways that explain the overexpression of these genes as a result of PDT remain unclear, these may indicate that there are a set of genes that are related to response to PDT [244]. In the same way, further characterization of mechanisms of cellular uptake and death still lack. Additionally, we should also look for a description of the functional changes induced by photooxidation of biomolecules, as well as for establishing relationships between products of photooxidation to biological outcomes.

## 5. Conclusions

We expect to have offered an overview on the processes taking place in PDT with a target-based approach, tracing relationships between processes happening at molecular and biological levels. It is evident that many benchmarks are still missing and we hope that this review sheds light on important challenges and open questions still remaining in this field.
